# Mid-gut ACTH-secreting neuroendocrine tumor unmasked with ^18^F-dihydroxyphenylalanine-positron emission tomography

**DOI:** 10.1530/EDM-14-0104

**Published:** 2015-03-01

**Authors:** Julien Ducry, Fulgencio Gomez, John O Prior, Ariane Boubaker, Maurice Matter, Matteo Monti, Yan Pu, Nelly Pitteloud, Luc Portmann

**Affiliations:** 1 Services of Endocrinology Diabetes and Metabolism, Lausanne University Hospital, Lausanne, Switzerland; 2 Nuclear Medicine, Lausanne University Hospital, Lausanne, Switzerland; 3 Visceral Surgery, Lausanne University Hospital, Lausanne, Switzerland; 4 Internal Medicine, Lausanne University Hospital, Lausanne, Switzerland; 5 Institute of Pathology, Lausanne University Hospital, Lausanne, CH-1011, Switzerland

## Abstract

Ectopic ACTH Cushing's syndrome (EAS) is often caused by neuroendocrine tumors (NETs) of lungs, pancreas, thymus, and other less frequent locations. Localizing the source of ACTH can be challenging. A 64-year-old man presented with rapidly progressing fatigue, muscular weakness, and dyspnea. He was in poor condition and showed facial redness, proximal amyotrophy, and bruises. Laboratory disclosed hypokalemia, metabolic alkalosis, and markedly elevated ACTH and cortisol levels. Pituitary was normal on magnetic resonance imaging (MRI), and bilateral inferior petrosal sinus blood sampling with corticotropin-releasing hormone stimulation showed no significant central-to-periphery gradient of ACTH. Head and neck, thoracic and abdominal computerized tomography (CT), MRI, somatostatin receptor scintigraphy (SSRS), and ^18^F-deoxyglucose-positron emission tomography (FDG-PET) failed to identify the primary tumor. ^18^F-dihydroxyphenylalanine (F-DOPA)-PET/CT unveiled a 20-mm nodule in the jejunum and a metastatic lymph node. Segmental jejunum resection showed two adjacent NETs, measuring 2.0 and 0.5 cm with a peritoneal metastasis. The largest tumor expressed ACTH in 30% of cells. Following surgery, after a transient adrenal insufficiency, ACTH and cortisol levels returned to normal values and remain normal over a follow-up of 26 months. Small mid-gut NETs are difficult to localize on CT or MRI, and require metabolic imaging. Owing to low mitotic activity, NETs are generally poor candidates for FDG-PET, whereas SSRS shows poor sensitivity in EAS due to intrinsically low tumor concentration of type-2 somatostatin receptors (SST2) or to receptor down regulation by excess cortisol. However, F-DOPA-PET, which is related to amine precursor uptake by NETs, has been reported to have high positive predictive value for occult EAS despite low sensitivity, and constitutes a useful alternative to more conventional methods of tumor localization.

**Learning points:**

- Uncontrolled high cortisol levels in EAS can be lethal if untreated.
- Surgical excision is the keystone of NETs treatment, thus tumor localization is crucial.
- Most cases of EAS are caused by NETs, which are located mainly in the lungs. However, small gut NETs are elusive to conventional imaging and require metabolic imaging for detection.
- FDG-PET, based on tumor high metabolic rate, may not detect NETs that have low mitotic activity. SSRS may also fail, due to absent or low concentration of SST2, which may be down regulated by excess cortisol.
- F-DOPA-PET, based on amine-precursor uptake, can be a useful method to localize the occult source of ACTH in EAS when other methods have failed.

## Background

Ectopic ACTH Cushing's syndrome (EAS), accounting for 5–10% of all cases of Cushing's syndrome, is mainly caused by neuroendocrine tumors (NETs) of lungs, pancreas, thymus, or tumors arising in other, less frequent sites (1). After ruling out a pituitary origin, the task to localize the source of ACTH can be challenging due to small tumor size and lack of specificity of computed tomography (CT)-scan and magnetic resonance imaging (MRI). Thus, diagnostic modalities based on tumor biology need to be used.

As epithelial-derived tumors arising in different organs and displaying predominant neuroendocrine differentiation, NETs share both organ-characteristic and neuroendocrine features (2). From a clinicopathological stand point, they range from benign neoplasms to rapidly progressing hormone-secreting carcinomas, and from occult to large symptomatic tumors (3). Functional diagnostic modalities include: i) somatostatin receptor scintigraphy (SSRS) with ^111^Indium-Pentetreotide or with ^68^Gallium-DOTA-TATE-PET takes advantage of the high concentration of type 2 somatostatin receptors (SST2) that is commonly observed in NETs; ii) 6-[fluoride 18]fluoro-dihydroxyphenylalanine-positron emission tomography (F-DOPA-PET), based on the ability of APUD-derived NETs to take up, accumulate, and decarboxylate amine precursors to synthesize, store, and secrete bioactive substances; and iii)^ 18^F-deoxyglucose-PET (FDG-PET) scan that aims at imaging glycolytic activity in tumors that have an elevated metabolic rate (4).

Notwithstanding a frequently indolent course, NETs display a marked tendency to metastasize and progress (5). Metastatic tumors may require focal treatment (e.g. thermal ablation or chemoembolization) or systemic medical therapy. Somatostatin receptor-targeted radiolabeled peptides, and therapies based on the inhibition of tyrosine kinase or mammalian target of rapamycin, have proven efficacy to slower disease progression (6) (7) (8) (9) (10). Cytotoxic chemotherapy can also be used in patients with progressive metastatic disease and no other treatment option (11). In addition, somatostatin receptor analogues often result in symptomatic relief by hampering hormone secretion in functional NETs (6). However, at present time only complete surgical removal offers long-term remission and cure. Therefore early localization of newly diagnosed NETs is crucial. In the particular case of EAS, the intense adrenal response to sustained ACTH may favor early recognition of the syndrome, but does not necessarily facilitate the localization of a small responsible tumor. The patient described in this case report presented with EAS caused by a small ACTH-secreting NET in an unusual location.

## Case presentation

A 64-year-old man, newly diagnosed with hypertension, presented with intense fatigue, muscular weakness, and shortness of breath (NYHA Stage IV), which rapidly increased within 4 months. On examination, the patient was in poor physical condition but hemodynamically stable without respiratory insufficiency. He showed slight facial redness, marked proximal limb muscles atrophy, and mild skin thinning with bruises. There was no moon face, purple striae, supra-clavicular fat pads, or buffalo hump. Laboratory data revealed severe hypokalemia (K 1.7 mmol/l) with metabolic alkalosis (base excess 10.9 mmol/l).

## Investigation

### Confirmation of Cushing's syndrome

Random plasma cortisol was 1600 nmol/l (normal range at 1700 h: 40–250 nmol/l), with ACTH 261 ng/l (normal range at 1700 h: 5–35 ng/l), and urinary free cortisol 9181 nmol/day (normal range: 20–220 nmol/day). Plasma cortisol level on a 2-day high-dose dexamethasone suppression test (2 mg/6 h) decreased from 1221 to 721 nmol/l.

### Investigation of ACTH-dependent Cushing's syndrome

Pituitary-centered MRI showed no evidence of disease especially in hypothalamus or pituitary gland. Bilateral inferior petrosal sinus blood sampling (BIPSS), including corticotropin-releasing hormone (CRH) stimulation, showed no significant central-to-periphery ACTH gradient and ruled-out a pituitary origin of ACTH excess (Table 1).

**Table 1:** Bilateral inferior petrosal sinus sampling (BIPSS) results. Plasma cortisol and ACTH levels before and after CRH injection, 1 μg/kg in i.v. bolus

	**Time** (min)								
**−15**	**0**	**5**	**10**	**15**	**25**	**35**	**50**	**60**
Peripheral vein: ACTH (ng/l)	195	223	228	229	229	207	204	184	187
Peripheral vein: cortisol (nmol/l)	1018	983	946	990	951	1037	1054	951	899
IPS left: ACTH (ng/l)	215	259	249	254	252	237	225	207	208
IPS right: ACTH (ng/l)	226	257	274	277	281	257	244	222	241

### Tumor localization of EAS

**Figure 1:**
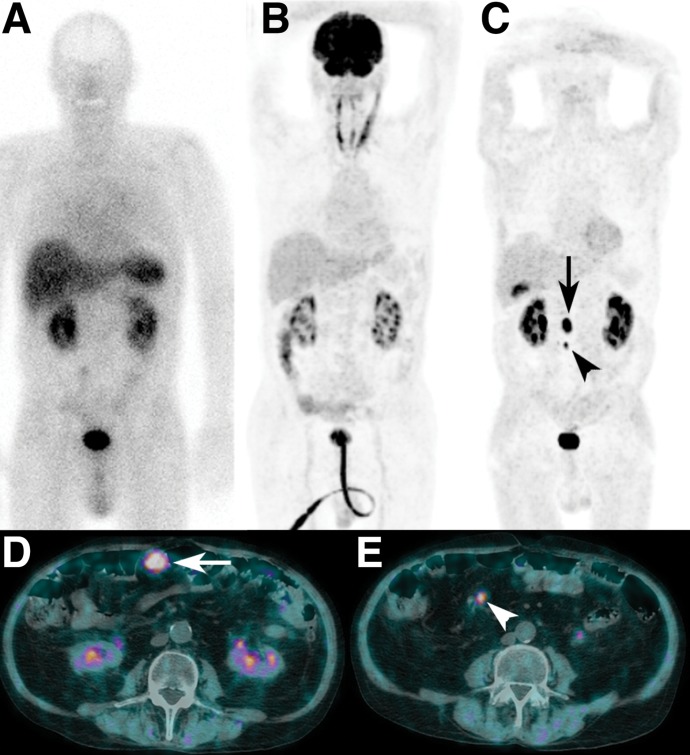
(A) ^111^In-octreotide scintigraphy and (B) ^18^FDG-PET did not reveal any pathological tracer uptake. (C) ^18^F-DOPA PET-CT allowed to locate a primary jejunal tumour (D) (arrow) and revealed metastatic lymph node(s) (E) (arrowhead).

Head and neck, thorax, and abdomen 3 mm-sliced CT-scan and MRI were unremarkable, excepted for moderately hyperplasic, nonnodular adrenals; SSRS (Fig. 1A) and FDG-PET/CT (Fig. 1B) failed to localize any functionally active lesion; finally, F-DOPA-PET/CT scan disclosed a 20 mm active tumor in the jejunum wall with a possible adjacent metastatic lymph node (Fig. 1C, D and E).

## Treatment

### Surgical treatment

At laparotomy, a segmental resection of the mid-jejunum was performed, including adjacent mesenteric lymphadenectomy. Histopathology revealed two adjacent well-differenced NETs in the jejunum wall, displaying low proliferation index (one mitosis per ten high-power fields; Ki67 1%), classified as WHO and ENETS grade 1 (3). The largest tumor (2 cm) invaded the whole jejunum wall up to the serosa, and was associated with a local peritoneal tumor deposit and with three metastatic lymph nodes out of five, showing capsular invasion (TNM: pT4 pN1 (3/5) M1 G1 R0) (3). The immunohistochemistry showed ACTH expression in 30% of the cells. The adjacent 0.5 cm tumor invaded the muscularis mucosae only and showed no vascular invasion and no ACTH expression.

## Outcome and follow up

Soon after surgery, ACTH and cortisol fell to low levels and transient adrenal insufficiency required cortisol replacement. The postoperative hospital stay was complicated on day 9 by a lower limb deep venous thrombosis and pulmonary embolism. Following anticoagulation, the patient was discharged uneventfully 5 days later.

The patient fully recovered, and after a follow up of 26 months, ACTH and cortisol levels remain normal and sequential CT scans show no evidence of recurrence.

## Discussion

Cushing's syndrome as paraneoplastic syndrome is a well-known phenomenon associated with NETs. ACTH-secreting tumors are more commonly encountered with bronchial carcinoids and small cell lung cancer (12).

Small ACTH-secreting tumors may remain undetected on anatomic imaging (pituitary MRI, and neck and trunk CT and MRI), despite clinical and biochemical evidence of ACTH-dependent Cushing's syndrome, and noninvasive dynamic tests are not always able to distinguish between Cushing's disease and EAS due to NETs. In our case, the intermediate cortisol suppression of 41% on high-dose dexamethasone was consistent with either possibility, and BIPSS was necessary to confirm EAS.

Surgery is a key element in therapy, allowing radical resection of metastases-prone NETs and in the setting of EAS rapid control of life-threatening cortisol excess, which can avoid chemical adrenolysis or palliative surgical adrenalectomy (13). Small abdominal NETs causing EAS are, however, especially difficult to localize using anatomical imaging techniques, and use of metabolic modalities may be required. Jejunum location is extremely rare among small bowels NETs causing EAS (14) (15) (16).

SSRS, which was negative in our case, is expected to identify NETs expressing high concentrations of cell membrane SST2, for which octreotide displays selective high-binding affinity. However, the sensitivity of SSRS in EAS is relatively low, ranging from 25 to 60% (1) (13) (17), suggesting low receptor expression in this syndrome compared with other NETs. This could be an intrinsic characteristic of tumors causing EAS, but a functional mechanism consisting of selective downregulation of SST2 induced by elevated cortisol, can also been advocated. In patients with primary adrenal insufficiency, somatostatin administration resulted in more effective ACTH suppression in patients left on low cortisol than on cortisol replacement (18); in patients with Nelson's syndrome, octreotide was more efficient to lower ACTH, especially after temporary withdrawal of cortisol replacement, than in patients with Cushing's disease and high cortisol levels (19); and exposure to dexamethasone decreases the *in vitro* expression of *SST2*-mRNA in pituitary adenoma cells (20). In this context, we hypothesize that targeting the SST with pasireotide, a ligand with high affinity for SST5, might result in better sensitivity for occult EAS, knowing that the expression of *SST5* mRNA in pituitary adenoma corticotrophs is five- to ten-fold higher than that of *SST2* mRNA (21). Indeed, pasireotide is more efficient than octreotide to control *in vitro* ACTH in the presence of high levels of glucocorticoid (20), and was proven to significantly decrease urinary cortisol excretion in patients with Cushing's disease (22). However, a similar expression of SST5 in ectopic ACTH tumors and a similar efficacy of pasireotide to treat EAS remain to be proven. On the other side, SSRS using ^68^Gallium peptide receptor radionucleides with octeotide derivatives were superior to ^111^Indium-Pentetreotide for NET detection (23), and ^68^Ga-DOTA-TATE-PET, using octreotate, showed a high sensitivity to detect well-differentiated metastatic NETs (24). However, to our knowledge these radionucleides have not been tested for occult EAS.

FDG-PET may highlight tumors with high metabolic activity, but shows a low sensitivity (35%, CI 15–61%) to identify NETs causing EAS (25). This examination was negative in our case, possibly because of the low proliferation index of the tumors.

F-DOPA-PET imaging is based on the ability of neuroendocrine cells to take up, accumulate, and decarboxylate amine precursors for the synthesis of bioactive substances. It has emerged as a diagnostic and staging tool for NETs, and was of utmost importance to solve our case. Furthermore, based from our case it can be hypothesized that the tumor with hormone secretion required more precursor uptake than the inactive one, possibly through upregulated membrane transport systems: this would explain why the ACTH-secreting tumor and its adjacent lymph-node metastases were accurately identified with F-DOPA-PET, whereas the neighboring nonsecreting NET remained silent, despite the fact that both tumors had similarly low mitotic indexes.

Zemskova *et al*. (26) showed that of the 11 patients with proven EAS but negative CT, MRI, and SSRS, the F-DOPA-PET identified tumors in six patients, all of whom had intra-thoracic NETs. This 55% sensitivity is lower than the 69–96% sensitivity described for midgut or hindgut carcinoids with no EAS (27) (28) (29), and suggests that carcinoids take up DOPA more efficiently than nonsecreting or ACTH-secreting NETs. However, the per-tumor-positive predictive value of F-DOPA-PET in the small series of intra-thoracic EAS was 100% (eight of eight tumors) (26). We now show that F-DOPA-PET may constitute a useful alternative to unveil occult EAS also in the less frequent small intestine location.

## Conclusion

The optimal therapy of EAS remains surgical excision of secreting tumor(s). However, small tumors causing EAS are difficult to detect on anatomical imaging and may require metabolic imaging techniques. We describe the case of a 64-year-old man presenting an occult EAS. The source of the secretion remained undisclosed after CT-scan, MRI, FDG-PET and SSRS. A small NET tumor causing EAS has been finally unveiled with FDOPA-PET. Despite its relatively low sensitivity, F-DOPA-PET should be considered as a useful alternative to locate elusive tumors in occult EAS when all other diagnostic modalities have failed.

## Patient consent

Written informed consent has been obtained from the patient for publication of the case report and accompanying images.
